# Arctic freshwater impact on the Atlantic Meridional Overturning Circulation: status and prospects

**DOI:** 10.1098/rsta.2022.0185

**Published:** 2023-12-11

**Authors:** Thomas W. N. Haine, Ali H. Siddiqui, Wenrui Jiang

**Affiliations:** Earth and Planetary Sciences, Johns Hopkins University, Baltimore, MD 21218, USA

**Keywords:** Arctic Ocean freshwater, sub-polar North Atlantic Ocean, Atlantic Meridional Overturning Circulation

## Abstract

Arguably, the most conspicuous evidence for anthropogenic climate change lies in the Arctic Ocean. For example, the summer-time Arctic sea ice extent has declined over the last 40 years and the Arctic Ocean freshwater storage has increased over the last 30 years. Coupled climate models project that this extra freshwater will pass Greenland to enter the sub-polar North Atlantic Ocean (SPNA) in the coming decades. Coupled climate models also project that the Atlantic Meridional Overturning Circulation (AMOC) will weaken in the twenty-first century, associated with SPNA buoyancy increases. Yet, it remains unclear when the Arctic anthropogenic freshening signal will be detected in the SPNA, or what form the signal will take. Therefore, this article reviews and synthesizes the state of knowledge on Arctic Ocean and SPNA salinity variations and their causes. This article focuses on the export processes in data-constrained ocean circulation model hindcasts. One challenge is to quantify and understand the relative importance of different competing processes. This article also discusses the prospects to detect the emergence of Arctic anthropogenic freshening and the likely impacts on the AMOC. For this issue, the challenge is to distinguish anthropogenic signals from natural variability.

This article is part of a discussion meeting issue ‘Atlantic overturning: new observations and challenges’.

## 1. Introduction

An essential challenge in physical oceanography and climate dynamics concerns the influence of polar low-salinity seawater on the global ocean circulation. At low latitudes, the ocean loses water to the atmosphere because evaporation exceeds precipitation. The atmosphere carries this water polewards where it accumulates in the surface ocean and thereby decreases salinity. This low-salinity seawater (‘freshwater’ in the parlance of the field) is carried equatorwards by the ocean circulation, which replenishes the low-latitude ocean and completes the cycle. The equatorwards flow of freshwater affects the circulation itself, however, because the low-salinity water has low density and therefore inhibits vertical exchange [1].

These processes occur prominently in the sub-polar North Atlantic Ocean (SPNA), where low-salinity outflow from the Arctic Ocean impinges on regions of strong vertical exchange. This strong vertical exchange forms a branch of the Atlantic Meridional Overturning Circulation (AMOC), which plays a leading role in North Atlantic and northern hemisphere climate (see [2,3] and the references therein).

Research in the last 30–40 years has established that this system is changing. The system fluctuates spontaneously over years and decades, and it changes in response to exogenous (anthropogenic) climate forcing. Specifically, extensive sustained efforts to observe and model Arctic Ocean processes have revealed large, interannual, near-surface Arctic freshwater anomalies. These anomalies appear to have natural origins, with anthropogenic decadal trends superimposed (these issues are discussed in §2). It is also known that, historically, freshwater from the Arctic propagates to the SPNA as a continuous stream with large anomalies. Furthermore, coupled climate models project that this Arctic Ocean freshwater export to the SPNA will increase in the twenty-first century. Extensive, sustained efforts to observe and model SPNA processes have also revealed large, interannual, near-surface SPNA salinity (freshwater) anomalies. These anomalies appear to have natural origins, with no clear role for anthropogenic forcing (§3). Observed anomalies in the AMOC also appear to be natural (§4). Moreover, coupled climate models project that the AMOC will weaken in the twenty-first century. Yet it is unclear when, and in what way, the Arctic anthropogenic freshening signal will be detected in the SPNA (§5) and how it will impact the SPNA stratification and circulation, and the AMOC (§6).

This article reviews and synthesizes the literature on these issues. The specific goals are to characterize the historical Arctic Ocean and SPNA salinity variations and discuss their mechanisms. The approach is empirical and quantitative. The approach is synthetic, in the sense that it tries to summarize the state of knowledge and speculate about future prospects. It also focuses on basin-scales (from 100s to 1000s of km) and long periods (from years to decades). The article focuses on how the Arctic Ocean affects the SPNA, and hence the AMOC, not the other way round. It also focuses on oceanic processes, not atmospheric or coupled ocean/atmosphere processes. The article concludes by articulating the present gaps in understanding on how Arctic freshwater impacts the SPNA and the AMOC, and on the causes of AMOC fluctuations. A strategy to close these gaps is outlined.

Although the article is mainly a review and synthesis, some new analyses are presented from a dynamical state estimate from an ocean circulation model (ECCOv4r4; see §7a) and from a gridded data synthesis (EN4; see §7b). The new results confirm, extend and synthesize the results from published papers. They allow us to construct a coherent synthesis of the impact of historical Arctic freshwater anomalies on the SPNA, at least in ECCOv4r4. Such a view does not exist in the published literature. In turn, this ECCOv4r4 synthesis motivates the open questions and recommendations in §6.

## 2. Arctic Ocean freshwater variations and mechanisms

**Figure 1.**
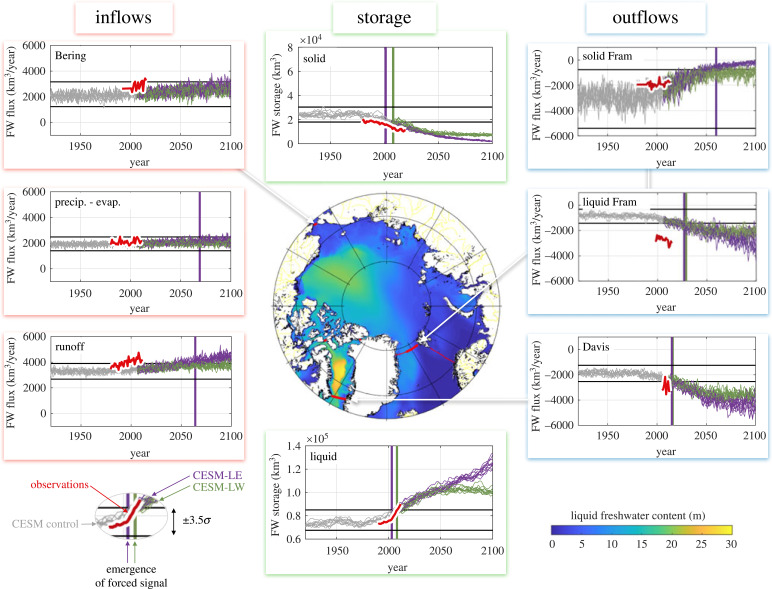
Observations (in red) and climate model projections of the Arctic Ocean freshwater cycle. The left (right) subplots show the principal time series of freshwater (FW) inflows (outflows; ${\mathrm{km}}^{3}\;{\mathrm{yr}}^{-1}$, relative to $S_{\text{ref}}=34.8\;g\;{\mathrm{kg}}^{-1}$; positive freshens the Arctic Ocean). The middle subplots show the freshwater volume stored in the Arctic Ocean as sea ice (solid, top) and liquid (bottom) freshwater (${\mathrm{km}}^{3}$ relative to $34.8\;g\;{\mathrm{kg}}^{-1}$). Results from the CESM historical control (grey), large ensemble (LE, purple) and low warming (LW, green) experiments are shown (from [9]). The subplots show when the forced, anthropogenic signal emerges (the time of first permanent departure from the ±3.5σ envelope of control variability, where σ is the standard deviation; horizontal and vertical lines). The basemap shows the liquid freshwater content, which is the vertically integrated salinity anomaly relative to $34.8\;g\;{\mathrm{kg}}^{-1}$. Adapted from Haine [10].

Observations show freshwater accumulating in the Arctic Ocean in the last few decades [4]. The first reliable estimate of the liquid freshwater content (LFC) of the Arctic Ocean was $97\;000\;{\mathrm{km}}^{3}$ [5]. (LFC is the integrated, normalized salinity anomaly relative to a reference salinity of, in this case, $S_{\text{ref}}=34.8\;g\;{\mathrm{kg}}^{-1}$.) Several studies have updated this value to quantify the freshwater accumulation over time. For example, Rabe *et al.* [6] estimate an extra $12\;000\;{\mathrm{km}}^{3}$ over 1992–2012. Haine *et al.* [7] estimate an extra $5300\;{\mathrm{km}}^{3}$ for 2000–2010 relative to 1980–2000. Proshutinsky *et al.* [8] estimate an extra $6400\;{\mathrm{km}}^{3}$ of liquid freshwater between 2003 and 2018 in the Beaufort Gyre, which is the largest Arctic freshwater reservoir. This build-up of liquid freshwater is shown in figure 1 (Liquid Storage panel), which shows observations of liquid freshwater volume increasing (red line; see [7,10] for full discussion and details on the data sources; and see [11] for a recent update). Figure 1 reveals the sources of the extra liquid freshwater too: they are reduced sea ice (Solid Storage panel), increased runoff and increased inflow through Bering Strait (left hand panels). The observed outflows (right hand panels) are unchanged or increasing in magnitude (Liquid Fram Strait panel; recent observations of Fram Strait liquid freshwater flux show no overall increase [12]). They do not match the increased inflows, however, causing the freshwater accumulation in the Arctic Ocean.

Figure 1 also shows results from the Community Earth System Model (CESM) version 1.1 based on Jahn & Laiho [9]. The CESM is a fully coupled, state-of-the-art global Earth system model [13]. The model results comprise an ensemble of historical control simulations (grey) and two ensembles of the twenty-first century projections (the large ensemble in purple and low warming scenario in green; see [9,14,15] for details). Using the amplitude of the control ensemble variability (horizontal lines) allows to determine when the anthropogenic-forced signals emerge (vertical purple and green lines; see [9] for details). The anthropogenic decline in Arctic sea ice emerged first in the 2000s (Solid Storage panel [16,17]). The anthropogenic increase in Arctic liquid freshwater emerged next in the 2010s (Liquid Storage panel). None of the inflow or outflow fluxes in figure 1 show emergence of an anthropogenic signal yet. The CESM results suggest that anthropogenic effects will increase the freshwater flux through Davis Strait, however, with a signal emerging in the 2020s. The Fram Strait fluxes are projected to change too, with less solid (sea ice) flux, more liquid flux (and more total flux), but the anthropogenic-forced signal is not expected to emerge for 15–40 years.

The CESM results match the observations in figure 1 reasonably well, although the CESM liquid Fram Strait freshwater flux is too small. In recent follow-up studies, Zanowski *et al.* [18] and Weijer *et al.* [19] show that other Coupled Model Intercomparison Project 6th phase (CMIP6) coupled climate models do not have this bias. Still, more work is needed to characterize the fingerprint of anthropogenic perturbation to the Arctic freshwater cycle in the coming decades. The projected increase in atmospheric moisture flux convergence is moderately well established [20–23], but the anticipated changes to the marine outflows are poorly known. Moreover, well-known, stubborn biases exist in the Arctic Oceans of CMIP6 models [11,24–27].

This evidence focuses on the *kinematic* inflows and outflows of freshwater to the Arctic (meaning they do not involve circulation changes). But *dynamical mechanisms* (involving circulation changes) are also important, especially for the Beaufort Gyre. Proshutinsky *et al.* [8] summarize three main factors controlling the freshwater build-up in the Beaufort Gyre:

(i) Ekman pumping from anticyclonic winds, which accumulates freshwater from around the gyre, including runoff from the shelves, and deepens the halocline [28–31].
(ii) Ice melt and growth, which limits the gyre spin-up. This ‘Ice-Ocean Governor’ feedback mechanism emphasizes the role of sea ice in controlling geostrophic currents [32]. Specifically, the surface ocean stress depends on the difference between the sea ice velocity and the surface ocean velocity. Therefore, stress on the ocean can change, hence changing Ekman pumping and freshwater accumulation, by changing sea ice conditions with fixed winds [33,34].
(iii) Stratification and mixing changes along continental slopes, which deepens the halocline and lengthens the gyre spin-up time [35,36].

In particular, the Beaufort Gyre circulation has strengthened (become more anticyclonic) and expanded as the liquid freshwater has accumulated over the last 30 years [37]. This strengthening is associated with stronger sea level air pressure (SLP) over the western Arctic [38]. Weak Beaufort Gyre circulation events (and weak sea SLP) have also occurred, however, for example in 1989 [7]. Modulating the Beaufort Gyre strength by varying the western Arctic wind field (i.e. SLP) triggers large flushing of Arctic freshwater to the SPNA both east and west of Greenland, at least in model experiments [29,39]. Therefore, concern exists that the Arctic Ocean is primed to release freshwater to the SPNA, either in flushing events or as a steadily freshening stream.

In summary, observations show freshwater accumulating in the Arctic Ocean in the last few decades. Coupled climate models attribute this freshwater accumulation to anthropogenic forcing. Although understanding of the mechanisms responsible for the accumulation is incomplete, evidence suggests that a shift in Arctic Ocean winds could trigger a flushing of this freshwater into the North Atlantic.

## 3. Sub-polar North Atlantic freshwater variations and mechanisms

**Figure 2.**
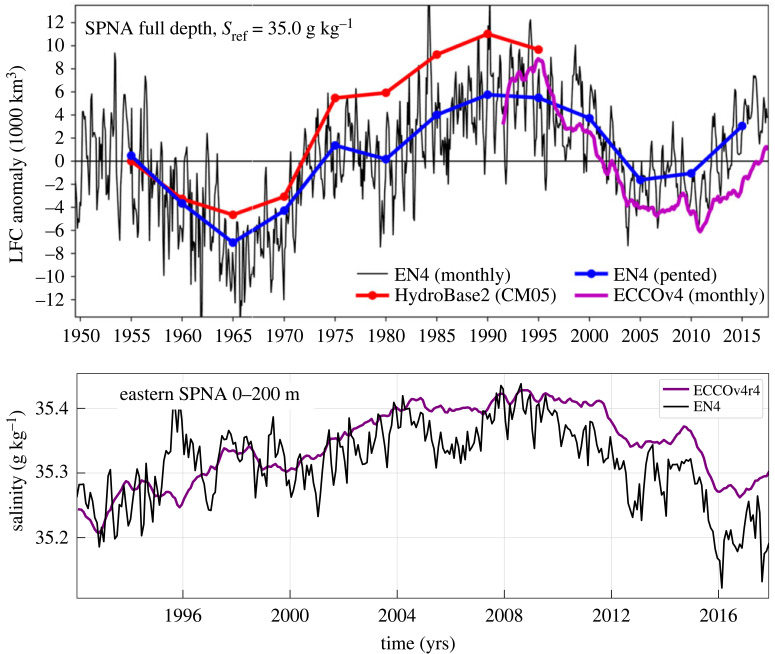
Diagnostics of sub-polar North Atlantic (SPNA) salinity variations. Upper: LFC anomaly relative to $S_{\text{ref}}=35.0\;g\;{\mathrm{kg}}^{-1}$ for the SPNA. Data are from the EN4 climatology (monthly and 5-year mean [40]), the HydroBase2 climatology [41] and ECCOv4r3. Adapted from Tesdal & Haine [42]. Lower: Average salinity for the upper 200 m of the eastern SPNA from the EN4 climatology, and the ECCOv4r4 state estimate (§7a). See figure 3 for the definition of the eastern SPNA region.

Observations show large-scale freshening events in the SPNA on decadal time scales. For example, figure 2 shows the liquid freshwater content for the SPNA since 1950 from hydrographic climatologies and the ECCOv4r3 dynamically consistent state estimate (§7a). The liquid freshwater content estimates broadly agree and show decade-long freshening events starting around 1965, 1980 and 2010. These events have been called ‘Great Salinity Anomalies’ (GSAs) [43–46]. They involve changes in liquid freshwater content of around $10\;000\;{\mathrm{km}}^{3}$, which is similar to the changes seen in the Arctic freshwater reservoirs in figure 1. GSAs appear to be a natural mode of Arctic/Atlantic Ocean variability that have occurred for at least the last century [47–49].

**Figure 3.**
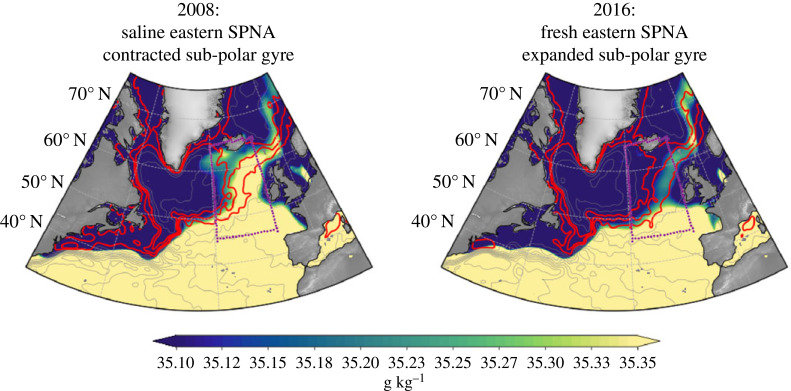
Variations in eastern SPNA salinity modulated by the NAC. Colours show annual-average surface salinity [from EN4; 40] for 2008 (2016), which correspond to saline (fresh) years in the eastern SPNA (purple box). Contours show the average sea level (absolute dynamic topography from AVISO) for the preceding two years (2006–2007 and 2014–2015), which correspond to contracted and expanded sub-polar gyre states. The contours are from −0.8 to 0.8 m with a spacing of 0.1 m and are smoothed with a 400 km Gaussian filter. The NAC follows the red contours (−0.3, −0.2 and −0.1 m) in the central North Atlantic. Adapted from Weijer *et al.* [19].

Figure 2 also shows the average salinity in the upper 200 m in the eastern SPNA over the last 30 years (see the purple boxes in figure 3 for the definition of the region). The data come from the EN4 observational climatology (§7b) and the ECCOv4r4 state estimate. Again, the data and state estimate broadly agree at interannual periods. The increase in liquid freshwater content for the whole SPNA centred on 2012 appears in the upper 200 m eastern SPNA as a shift from a salty anomaly in 2008 to a fresh anomaly in 2016 with a salinity change of around $0.2\;g\;{\mathrm{kg}}^{-1}$. Indeed, Holliday *et al.* [50] call 2014–2017 the largest freshening event in the eastern SPNA in the last 120 years.

The cause(s) of the 2016 fresh event (and of the 2008 saline event) are elucidated by the salinity and sea level observations in figure 3. The red sea level contours show the North Atlantic Current (NAC) path in the SPNA for the two years prior to the salinity anomalies (i.e. 2006–2007 and 2014–2015). Specifically, compare the red contours in the purple boxes in figure 3 for each period. In the two years before the 2008 saline anomaly, the NAC extended further to the west, shrinking the sub-polar gyre and allowing saline subtropical water to enter the eastern SPNA. In the two years prior to the 2016 fresh anomaly the NAC extended further to the east, expanding the sub-polar gyre and allowing fresh sub-polar water to enter the eastern SPNA. In other words, the upstream routing of saline subtropical or fresh sub-polar water determines the eastern SPNA salinity anomalies. The processes controlling eastern SPNA temperature anomalies are consistent: 2008 was a warm event, whereas 2016 was a cool event [42,51].

This argument is an example of a proximate mechanism to modulate the salinity in the eastern SPNA. Several ultimate causes for the salinity anomalies have been proposed in the literature. They include:

(i) The export of freshwater from the Arctic as sea ice and liquid freshwater via the Fram and Davis Straits to the western SPNA [39,49]. The fresh anomalies then propagate to the eastern SPNA in the NAC. For example, Holliday *et al.* [50] explain the 2016 fresh event as the rerouting of the Arctic-sourced Labrador Current water in the upper 200 m into the northern branch of the NAC.
(ii) Relatedly, saline events are attributed to anomalous salt transport from the subtropical gyre via the NAC [42,48,52–55].
(iii) Air/sea interaction in the SPNA. For example, Josey & Marsh [56] argue that the freshening from 1960 to 2000 can be largely explained by changes in the air–sea freshwater exchange, mainly increased precipitation.

**Figure 4.**
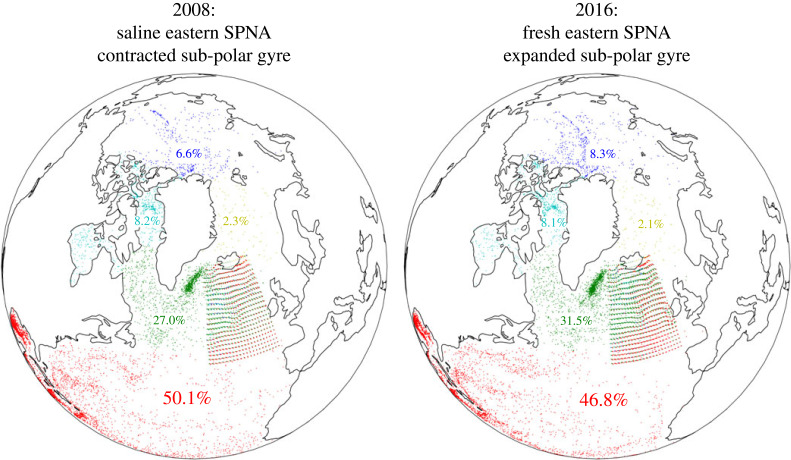
Three-dimensional Lagrangian particle origins in the ECCOv4r4 state estimate. The 7744 particles are released in the eastern SPNA on the grid of red dots over the upper 200 m in (left) 2008 and (right) 2016 when the eastern SPNA was saline (fresh) and the sub-polar gyre was contracted (expanded). The particles are backtracked for 16 years and coloured according to their starting region. The percentages show the fractions of the released particles from each starting region.

A natural and revealing complement to the Eulerian analyses in figures 2 and 3 is a Lagrangian perspective. A Lagrangian perspective emphasizes the complicated transport pathways that exist in reality, but that are hidden in the Eulerian LFC, salinity and sea level timeseries in figures 2 and 3. To this end, we show in figure 4 new results of three-dimensional backtracking Lagrangian particles in the ECCOv4r4 state estimate [57–60]. Particles are released from the eastern SPNA (upper 200 m) in 2008 and 2016 and integrated backwards for 16 years (§7c). The particles are coloured according to their source region 16 years before release. In both events, the regions that feed the eastern SPNA 16 years later are (in decreasing order of importance): the subtropical and tropical North Atlantic, the SPNA, the Arctic Ocean or the Canadian Arctic Archipelago (CAA) and the Nordic Seas. Most of the Arctic particles reach the SPNA via the transpolar drift and the CAA (west of Greenland) rather than via the Nordic Seas. Few particles reach the SPNA from the Beaufort Gyre over 16 years. The differences between the 2008 and 2016 events are as follows: There are 17% more particles from the SPNA in the 2016 event (meaning an increase from 27.0% of all particles to 31.5% of all particles, see figure 4, which is a 17% increase). There are 7% fewer from the subtropics and 27% more from the Arctic (9% more come from the Arctic, CAA and Nordic Seas combined).

In other words, before the 2016 fresh event: water resided longer in the SPNA being freshened by air/sea interaction, less saline water came from the subtropics and more freshwater came from the Arctic. These results are consistent with all of the mechanisms identified earlier.

Some potential mechanisms are considered to be less important. One example is anthropogenic loss of the Greenland Ice Sheet (GIS), which has not yet led to detectable SPNA freshening [61,62]. Nevertheless, uncertainty exists on the fate of GIS meltwater because it depends on circulation model resolution, and how the GIS discharge is parametrized [62–64]. These processes are not accurately represented in the ECCOv4r4 state estimate.

Finally, other studies emphasize *dynamical mechanisms* controlling eastern SPNA salinity. Wind and buoyancy fluctuations influence the circulation, especially on interannual and decadal timescales, respectively [65–68]. For example, when the North Atlantic Oscillation (NAO) is positive, anomalous mid-latitude westerly winds drive an expanded sub-polar gyre and fresh anomalies in the eastern SPNA, as in 2016 (figure 3 [19]). Conversely, when the NAO is negative, the sub-polar gyre contracts and saline anomalies occupy the eastern SPNA, as in 2008.

In summary, observations show decadal, upper ocean, propagating salinity variations in the SPNA since 1950. The salinity variations involve shifts in the NAC and expansion/contraction of the sub-polar gyre in the eastern SPNA. Understanding of the ultimate causes of the salinity variations is incomplete. Nevertheless, the leading candidate mechanisms are as follows: changes in salt transport from the subtropics and the Arctic, changes in the AMOC and changes in SPNA precipitation. These mechanisms are typically associated with changes in SPNA winds, especially the NAO.

## 4. Sub-polar North Atlantic AMOC variations

The AMOC has also been implicated in SPNA salinity anomalies. Observations show that the SPNA AMOC fluctuates on interannual to decadal periods. For example, it strengthened between 1980 and the mid-1990s, then weakened to the 2010s and is now possibly strengthening again [68,69]. These variations are attributed to atmospheric forcing, especially the winter NAO [65,66]. The variations broadly coincide with the fluctuations shown in figures 2 and 3. Indeed, Bryden *et al.* [70] estimate that the eastern SPNA freshening from 2008 to 2016 is consistent with the weakening of the $26^{\circ }\;N$ AMOC freshwater flux to the SPNA from 2009 to 2016. Robson *et al.* [71] found support for this idea in a coupled climate model.

Other studies emphasize the importance of the horizontal gyre circulation, instead of the AMOC, in controlling interannual to decadal SPNA variations. For example, Piecuch *et al.* [51] found in ECCOv4r3 that horizontal gyre circulation anomalies across the southern boundary of the SPNA mainly determine 1992–2015 SPNA heat content anomalies. Tesdal & Haine [42] reach the same conclusion for SPNA LFC anomalies. Both these studies consider anomalies for the entire, full-depth SPNA, however, integrating from the sea surface to the sea-floor. How this picture depends on different choices of control volume is unclear. For example, the salinity changes in the upper 200 m of the eastern SPNA seen in figure 2 may depend less on anomalies inherited from the subtropics (either from horizontal gyre circulation or AMOC changes). Moreover, Holliday *et al.* [72] use transbasin SPNA hydrographic sections to show that high heat flux associates with high AMOC strength, whereas high freshwater flux associates with high gyre circulation. Reconciling these divergent viewpoints is an important challenge.

Looking ahead to 2100, the AMOC is projected to decline in almost all coupled climate models as a result of anthropogenic forcing [73,74].^1^ Moreover, the AMOC may weaken irreversibly, meaning that the circulation system crosses a threshold (or tipping point) that leads to a nonlinear, abrupt slowdown [78]. This possibility is deemed to have low likelihood [17,79], but the impacts on humankind would be large [80,81]. Despite the possibility of such forced signals, the observed AMOC variations mentioned earlier are probably natural [82,83]. In other words, the anthropogenic-forced AMOC signal has not yet emerged from the noise of natural variability.

In summary, variations in both the AMOC and the horizontal gyre circulation have been implicated in SPNA salinity variations, based on evidence from both observations and models. Yet, inconsistencies remain, for example, to do with the importance of different circulation changes for different aspects of SPNA salinity. Although coupled climate models project AMOC weakening in the twenty-first century under anthropogenic climate change, the SPNA changes seen to date are probably natural.

## 5. Arctic/sub-polar North Atlantic salt exchanges

**Figure 5.**
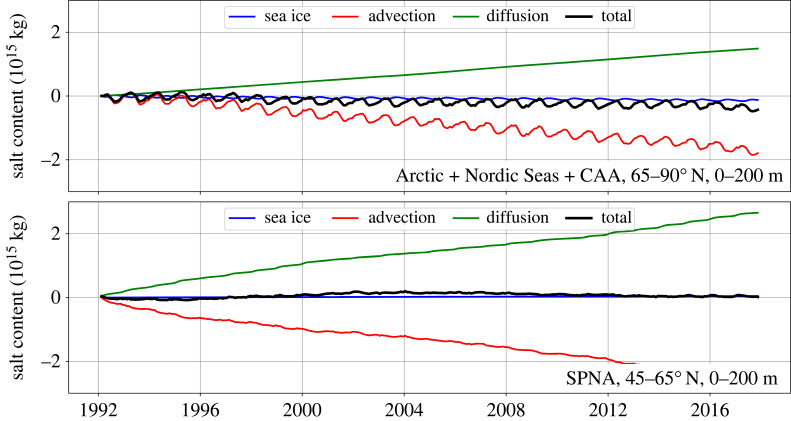
Decomposition of volume-integrated salt mass for the (upper) Arctic (north of 65${\;}^{\circ }$ N) and (lower) SPNA (45–65${\;}^{\circ }$ N) upper 200 m from the ECCOv4r4 state estimate. The cumulative (time-integrated) contributions to the total salt mass change due to advection, diffusion and sea ice are shown. For details on how each term is defined, see §7d.

Another useful perspective on the impact of Arctic freshwater export on the AMOC is the net exchange of salt between the Arctic and SPNA. Therefore, we examine this exchange in figure 5 using new results from ECCOv4r4 for 1992–2017. The SPNA is defined as the region between 45 and 65${\;}^{\circ }$ N, and the Arctic is defined as north of 65${\;}^{\circ }$ N (i.e. it includes the Nordic Seas and CAA). In both cases, only the upper 200 m of the water column is included. Figure 5 shows the cumulative (time-integrated) contribution of various processes to the change in the total mass of salt in these reservoirs (see §7d). These processes are as follows: advection across the faces of the reservoir, diffusion across the faces, and exchange with sea ice due to melting and freezing (sea ice has a salinity around $4\;g\;{\mathrm{kg}}^{-1}$). Note that there is no air/sea exchange of salt.

For the Arctic, Nordic Seas and CAA, figure 5 shows that diffusion increases the salt content (because the water is salty deeper than 200 m). Advection decreases the salt content (because the seawater outflow exceeds the seawater inflow by the water flux received from the atmosphere and land). Sea ice exchange also decreases the salt content (because, overall, the region exports salt in sea ice). Salt exchanges due to advection and sea ice have seasonal cycles. The effect on the total salt content is a decreasing trend over 1992–2017, which is due to an overall imbalance between sea ice, advection and diffusion.^2^ The total loss is about $0.35\times 10^{15}$ kg. This salt mass corresponds to an increase of about $10\;000\;{\mathrm{km}}^{3}$ of liquid freshwater relative to $S_{\text{ref}}=34.8\;g\;{\mathrm{kg}}^{-1}$ (assuming, reasonably, that the reservoir volume is constant). Thus, it is broadly consistent with the LFC increase discussed in §2.

For the SPNA, figure 5 shows that diffusion increases salt content and advection decreases it; again, the main salt balance is between these two terms. Sea ice is a weak factor for the SPNA, and no long-term trend is visible for the total salt content in figure 5.

**Figure 6.**
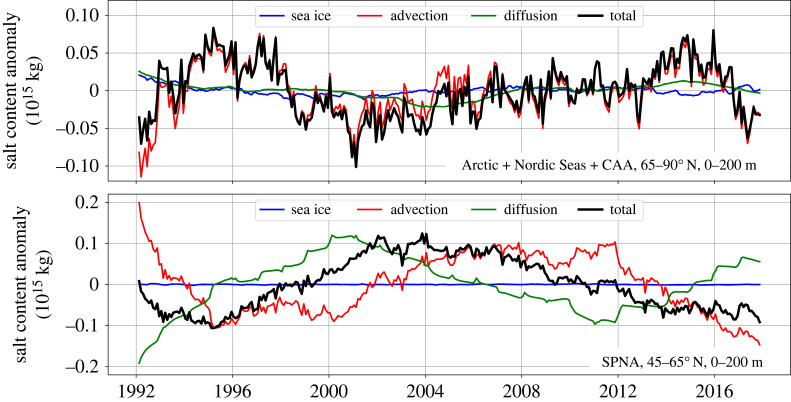
As shown in figure 5, except salt content anomalies are shown after removing linear trends. Note the y-axes differ between the two panels.

Interannual variations in the total salt content exist for both the Arctic and, especially, the SPNA in figure 5. These variations are shown in detail in figure 6, which shows the same timeseries with linear trends removed. For the Arctic, Nordic Seas and CAA, the variations have a magnitude of around $10^{14}\;\mathrm{kg}$ (corresponding to LFC variations of around $3000\;{\mathrm{km}}^{3}$). These variations are closely associated with variations in advection. For the SPNA, the variations have a similar magnitude, but they are associated with variations in both advection and diffusion. Salt content anomalies due to diffusion lead those due to advection, at least for the single fresh-to-salty-to-fresh cycle in ECCOv4r4 over 1992–2017.

The interannual SPNA salt content variations in figure 6 resemble the LFC variations seen in figure 2. The salt content minima in 1994 and 2016 correspond to the SPNA freshening events discussed in §3. Figure 6 shows that these freshening events were mainly associated with declining advection in ECCOv4r4. Diffusion counteracts them, but is weaker.

The contribution of advection to the SPNA salt anomalies in figure 6 is the sum of horizontal exchange across the two boundaries at 45${\;}^{\circ }$ N and 65${\;}^{\circ }$ N, and vertical exchange across 200 m. Of these terms, the advective flux across 45${\;}^{\circ }$ N is relatively large and is strongly anti-correlated with advective flux across 200 m (they nearly sum to zero; not shown). That means salt anomalies enter the SPNA control volume from the south, and mainly leave it by sinking across 200 m. This exchange resembles the AMOC in the SPNA. By contrast, advective salt flux anomalies across 65${\;}^{\circ }$ N are relatively smaller, by a factor of about four. The sum of the advective fluxes across 45${\;}^{\circ }$ N, 65${\;}^{\circ }$ N and 200 m (red line in figure 6) is relatively small compared with these individual advective terms. For the diffusive salt flux anomalies in figure 6 (green line), the flux across 200 m dominates.

Hence, for upper 200 m SPNA ECCOv4r4 salt anomalies, Arctic/SPNA salt exchange is an important (although subdominant) process alongside vertical exchange and horizontal exchange from the south. The role of the Arctic decreasing salt content trend shown in figure 5 (upper panel, black line) on the SPNA is unclear, however. Further study of the advective exchange across 65${\;}^{\circ }$ N is required to elucidate it, such as decomposing the net 65${\;}^{\circ }$ N flux into southbound Arctic salt import into the SPNA, and northbound export.

## 6. Summary, open questions and discussion

On the evidence from the published studies summarized earlier, and from the new results that provide a holistic context, the state of knowledge on freshwater variations in the Arctic and SPNA is as follows:

— Interannual Arctic freshwater fluctuations clearly exist, which appear to be natural. In addition, a decadal freshening trend exists, which appears to be anthropogenic.
— Arctic Ocean freshwater export to the SPNA is known to fluctuate naturally on interannual periods with several export anomalies thought to have occurred in the last 50–100 years.
— Interannual SPNA freshwater fluctuations (GSAs) clearly exist. They appear to be natural (not forced by anthropogenic effects), with no sign yet of a decadal freshening trend from the north. SPNA fresh anomalies seem to involve longer SPNA residence times, more Arctic water, and less subtropical water. Fluctuations in SPNA air/sea interaction and the AMOC are potentially important too. But the relative roles of these different processes, and their ultimate causes are still obscure.
— Climate model projections suggest that Arctic freshwater accumulation will continue, and Arctic freshwater export fluxes will increase in the twenty-first century, which will freshen the SPNA. Projections suggest that the anthropogenic freshening signal will emerge in the 2020s (Davis Strait, freshwater flux; Fram Strait ratio of liquid to solid freshwater fluxes).
— Climate model projections suggest that in the twenty-first century, the SPNA AMOC will weaken. There is a low-likelihood, high-impact possibility that the AMOC will weaken irreversibly.

In the light of this knowledge, some leading open questions are as follows:^3^

(i) When will Arctic anthropogenic freshening be detected in the SPNA?
(ii) What is the fingerprint of Arctic anthropogenic freshening in the SPNA and how will it be detected in the SPNA with the current observing network (if at all)?
(iii) When will Arctic anthropogenic freshening affect SPNA circulation?
(iv) What is the fingerprint of this circulation change and how will it be detected with the current observing network (if at all)?

To answer these questions on SPNA anthropogenic freshening, we require improved understanding of the mechanisms of SPNA salinity variability. Mechanistic understanding is essential to distinguish natural from anthropogenic variations (among several reasons) and to thus characterize the fingerprints of anthropogenic freshening. We hypothesize the following sequence of events: (a) The first Arctic anthropogenic SPNA freshening signals to emerge will be of small amplitude and therefore dynamically passive (not affect the circulation, namely a kinematic mechanism). (b) Dynamically active Arctic SPNA anthropogenic freshening signals will follow and will weaken the AMOC. As the initial dynamical freshening effects will be of small amplitude, they will affect the AMOC in a linear and, therefore reversible, way. (c) Any subsequent large amplitude Arctic SPNA anthropogenic freshening signals increase the risk of a nonlinear irreversible AMOC weakening. The implications of SPNA freshening on the AMOC in steps (b) and (c) also need to be better understood, especially as they pertain to climate impacts.

It is important to recognize that the SPNA may freshen due to anthropogenic effects that are unrelated to Arctic Ocean freshwater export, such as forced Greenland Ice Sheet melt [84] or forced changes to the NAO [85] or changes associated with anthropogenic aerosols [86]. It remains to be established if the AMOC weakening in (b) will be detectable with the present or future observing network (we know of no studies on this question). It is also possible that the AMOC will weaken for reasons other than an Arctic SPNA anthropogenic freshening signal.

To address the open questions (i)–(iv), the community should:

— Maintain the current observing network, such as the Arctic and SPNA hydrographic measurements and gateway flux observatories.^4^ No alternative method is known to observe the freshening signals.
— Expedite data dissemination, analysis and synthesis. In some cases, years have passed before data from *in situ* instruments have been processed and made public. Support is needed to facilitate and accelerate this pipeline.
— Extend and refine dynamically consistent reanalyses, such as ECCOv4r4. These state estimates are our best (albeit imperfect and provisional) tools to track and understand the basin-scale, decadal stratification and circulation changes.
— Study and refine coupled climate models to resolve Arctic Ocean biases, especially in the Atlantic Water, the halocline and the surface Polar Water layer, and thereby decrease the model spread in projected salinity changes [24,27].
— Perform consistent, robust budget analyses (like those in figures 2, 5 and 6). Some past studies have been plagued by ambiguities surrounding reference salinities [93,94]. Robust interpretation methods are now known, however [95], and should be universally adopted. Moreover, the sensitivity of budget analyses to choice of variable (LFC, salt), control volume (full-depth, upper ocean; whole SPNA, eastern SPNA) and data source (state estimates, circulation models) should be explored.
— Observe and understand SPNA freshwater dispersion. In particular, the processes controlling transport of Arctic freshwater off the Greenland and Canadian shelves into the deep SPNA occur at small space-time scales and are poorly observed, modelled and understood [62,96].
— Characterize the fingerprint of Arctic anthropogenic freshening in the SPNA and recommend strategies to observe it. An unprecedented opportunity exists to anticipate and observe fresh anomalies move through the system [97].

The aim of these activities is to elucidate the spread of Arctic anthropogenic freshening into the SPNA. They will establish the plausibility of the Arctic freshwater export process as an agent to change the SPNA, the AMOC and thereby contribute to the wider debate on SPNA anthropogenic change.

## 7. Methods

### (a) ECCO Ocean state estimate

The Estimating the Circulation and Climate of the Ocean (ECCO) state estimate is a solution to the Massachusetts Institute of Technology general circulation model (MITgcm; [98]). The solution is computed by fitting the MITgcm fields to several hundred million satellite (altimetry, sea surface temperature, sea surface salinity, gravimetry) and *in situ* (temperature, salinity) ocean observations for the period of satellite altimetry [99–101]. To produce the state estimate, the surface forcing, initial conditions and mixing coefficients are adjusted within their respective uncertainties. As the state estimate is a data-constrained solution to the free-running MITgcm, the solution is dynamically consistent, and it avoids unphysical nudges. Thus, closed, physically realistic salt budgets can by computed, such as in figures 5 and 6. In this article, we use ECCO version 4 release 4 (ECCOv4r4, [90,91,102]). The ECCOv4r4 solution is global and spans 1992–2017. The horizontal resolution is 1${\;}^{\circ },$ and there are 50 vertical levels whose thicknesses range between 10 m near the surface and 450 m near the bottom.

### (b) Ocean reanalysis: EN4

EN4 is a gridded global dataset for ocean temperature and salinity compiled by the United Kingdom Met Office ([40]; this paper also explains the origin of the ‘EN4’ name). It spans the period 1900 till present with quality control checks and bias removal corrections applied following Gouretski & Reseghetti [103]. We use EN.4.2.2 for our analysis.

### (c) Lagrangian particle analysis

The Lagrangian particle backtracking in figure 4 is performed using the seaduck open-source Python software, available at github.com/MaceKuailv/seaduck. The algorithm uses analytic formulae to compute the Lagrangian trajectories in three dimensions, assuming piecewise-constant-in time velocity fields and linear interpolation in space. The calculations use monthly averaged ECCOv4r4 velocity fields, but results are essentially unchanged if daily averaged velocity fields are used instead. Results are also essentially unchanged if the number of particles is increased by a factor of eight.

### (d) Salt budget analysis

The salt budgets shown in figures 5 and 6 are derived as follows. The equation for salinity S reads

7.1 $$\frac{\partial S}{\partial t}=-\nabla \cdot (uS)-\nabla \cdot F_{d}+F_{S},$$

where $F_{d}$ is the diffusive flux and $F_{S}$ is the salinity forcing due to salt exchange with sea ice. The other terms assume their conventional meanings. Note that no air/sea exchange of salt occurs. Integrating this equation over a fixed control volume V that is bounded by surface A gives

$$\int_{V}\frac{\partial S}{\partial t}\;dV=-\int_{A}u_{⊥}S\;dA-\int_{A}F_{d,⊥}\;dA+\int_{V}F_{S}\;dV,$$

where Gauss’ theorem has been applied to the divergent terms in (7.1) and the ⊥ subscript indicates the component perpendicular to surface A. Integrating over time yields the mass of salt, $M_{S}(t)$:

$$\begin{array}{rl} M_{S}(t)\equiv \rho_{0}\int^{t}\int_{V}\frac{\partial S}{\partial t^{\prime }}\;dV\;dt^{\prime } & \;=-\rho_{0}\int^{t}\int_{A}u_{⊥}S\;dA\;dt^{\prime }-\rho_{0}\int^{t}\int_{A}F_{d,⊥}\;dA\;dt^{\prime }+\rho_{0}\int^{t}\int_{V}F_{S}\;dV\;dt^{\prime }, \end{array}$$

where $\rho_{0}$ is the reference density of seawater. The four terms in this equation are called ‘total’, ‘advection’, ‘diffusion’ and ‘sea ice’ in figures 5 and 6.

## Data Availability

The ECCO datasets are publicly available on the SciServer system [104] and at podaac.jpl.nasa.gov/ECCO. The EN4 data are available at www.metoffice.gov.uk/hadobs/en4/.

## Footnotes

1 It is worth noting that some paleoclimate proxy data have been used to infer that the AMOC has weakened since the 1800s [75]. The claim is disputed, however [76], and a more complete analysis of proxy records is ambivalent on weakening [77].

2 One might ask which term is responsible for the overall decrease in Arctic, Nordic Seas and CAA salt content in figure 5. But the linear trends indicate that the individual salt fluxes are constant over 1992–2017. Therefore, the net salt loss cannot be attributed to any one of them: the fluxes simply sum to a constant negative value that indicates salt loss.

3 This list is, of course, incomplete and somewhat subjective. It focuses on the putative impacts of anthropogenic Arctic freshening on the SPNA and AMOC weakening, but other interesting questions abound.

4 For the gateway flux observations, see Wang *et al.* [11], and the references therein. For hydrographic observations, ice-tethered profilers [87] and the Argo program are particularly valuable. The Overturning in the Subpolar North Atlantic Program (OSNAP) is important for SPNA fluxes and hydrographic changes [88] . For a comprehensive framework to observe the Arctic Ocean, see Lee *et al.* [89]. For a comprehensive list of observations used to constrain ECCOv4r4, see Fukumori *et al.* [90,91]; the study by Nguyen *et al.* [92] is also useful.
